# Presence of Accessible Equipment and Interior Elements in Primary Care Offices

**DOI:** 10.1089/heq.2019.0006

**Published:** 2019-06-18

**Authors:** Nancy R. Mudrick, LeeAnn C. Swager, Mary Lou Breslin

**Affiliations:** ^1^ School of Social Work, Syracuse University, Syracuse, New York.; ^2^ Disability Rights Education and Defense Fund, Berkeley, California.

**Keywords:** disabilities, accessible equipment, primary care offices

## Abstract

**Purpose:** To describe the disability accessibility level of primary care office interiors and the presence of accessible examination equipment.

**Methods:** Data from on-site audits of 3993 primary care offices in California for 2013–2016 are descriptively analyzed. Architectural access is assessed using an instrument based on ADA Accessibility guidelines (ADAAG), along with noting accessibility of examination equipment.

**Results:** Compliance across architectural elements was ∼85%. Accessible examination tables and scales were observed in 19.1% and 10.9% of offices, respectively.

**Conclusions:** Proactive accessibility auditing makes visible the infrequent presence of accessible examination equipment. It offers data for tracking progress to increase medical office disability access.

## Introduction

We know physical and equipment characteristics in primary care offices constitute access barriers, affecting quality of health services and health disparities experienced by people with disabilities; we do not know the percentage of doctor offices that are accessible.^1–5^ Medical office physical accessibility includes parking, external entrances, public interior pathways, and arrangements and equipment inside the medical suite. Patient surveys and focus groups produce reports of accessibility problems, from distant parking to inaccessible examination tables and scales. Patients indicate delaying care for access reasons, including fear of injury during manual transfer from a wheelchair or other mobility device.^6–8^ We know less from providers. No national databases routinely collect and analyze site accessibility, although several states collect information for Medicaid provider ADA compliance.^9,10^ The limited number of provider studies, using surveys or direct observation of doctor offices, have revealed barriers in toilets and examination room size, a low percentage of offices with accessible examination equipment, and medical practices that refuse a referral because they cannot accommodate a patient with mobility impairments.^11–16^ These studies mostly have a small number of observations. Our prior analysis of primary care office audits in California 2006–2009 is the largest of these studies, with 2389 observations. We found that only 8.4% had a height adjustable examination table and 3.6% an accessible scale.^12^

The main objective of this brief report is to provide a descriptive update on medical office accessibility from recent on-site audits. These were collected from primary care offices in California 2013–2016 using a revised instrument, and a number of sites not in our previous study. Because providers are likely to have more control over interior elements of their offices, such as room and toilet configuration and examination equipment, than over the parking lot and public access elements of the building in which they are located, this analysis focuses on (1) the disability accessibility ratings of interior office elements and equipment and (2) associations between the presence of accessible examination equipment items and other interior elements.

## Data Source and Methods

In 2006 in California, a group of Medicaid Managed Care (MMC) plans began conducting physical site accessibility audits of their providers. Trained raters who work for the MMC plans conduct the audits. Where a number of practices are located in a large medical building, each practice is separately audited because interior office spaces differ. Raters are trained together on the use of the disability access audit instrument, although they are employed by different MMC plans. MMC plans audit a provider office when that practice joins the plan, and every 3 years thereafter. Health plans share audits to avoid duplicated visits to the same office because providers may be associated with more than one MMC plan. Thus, the same site can appear in the database of multiple plans.

The disability access audit tool has 86-items (a revised and expanded version of the 55-item audit instrument used from 2006–2010). The items are based upon the 2010 ADA Accessibility Guidelines (ADAAG) developed by the U.S. Access Board (www.access-board.gov), with five additional questions that ask about examination tables, patient lifts, and weight scales. The audit instrument requires assessment of parking and exterior path access; main entry, elevator, and interior path of travel; doctor office access in reception; signage for persons with visual or hearing limitation; toilet room characteristics; and examination room access, size, and equipment.

Recognizing that the audits covered a uniquely large set of offices, in 2010, we asked the plans if we could analyze their data. Five MMC plans sent us audits for 2006–2009, and we published descriptive findings on the physical accessibility of 2389 physician practices, including statistics on the presence of accessible examination tables and scales.^12^ In 2017, curious whether access had increased with recent attention to health care facility access, we again approached California MMC plans for their audit data. Five plans sent us data from audits conducted in 2013–2016. Four of the plans had sent us data in 2010; one plan was new to us. We received these data as five separate Excel spreadsheets, which we merged and cleaned. The state Site ID, zip code, and last audit date were used to eliminate duplicate entries in the merged dataset, producing 3993 observations. To our knowledge, this is the largest dataset of office audits.

Unfortunately, our dataset contains no additional information about the site, such as building size or age, or number of doctors and size of practice. This information would be useful for interpreting the findings. The five plans that sent us data do not cover California evenly. Eighty percent of the sites are in Southern California, with 52.5% from Los Angeles County. Only 2% of the observations are from Northern California with the remaining from the central part of the state, east, and west. We analyzed the 2013–2016 dataset as a cross-section to use all observations. Only some sites in the prior dataset are in the current one, and instrument wording differences make some comparisons problematic. IRB approval was not required. There are no human subjects; subjects are physical offices not identified by address.

## Results

Overall, accessible architectural elements are more likely to be present than accessible equipment. Table 1 shows compliance ranges from 76% to 99% for space in the reception area, clear path of travel through the medical suite, examination room door width and swing clearance, and lowered counter or alternative method for people to sign in or register. Some interior doors to a medical suite are fire doors, but where the door is not a fire door, 24% of office doors require more than 5 pounds of pressure to open. A smaller percentage (11.9%) of nonfire doors to the patient toilet require more than 5 lbs pressure to open. Once in the toilet room, challenges include the location of grab bars and toilet paper dispenser with respect to the accessible toilet. In 14.3% of bathrooms there are no grab bars or they are incorrectly located; 36.3% have toilet paper dispensers not correctly located. Among examination rooms, 16% are too small for someone using a wheelchair or scooter to enter and turn.

**Table 1.** Medical Office, Examination Room, and Examination Equipment Characteristics

**Medical office interior characteristic**	**% yes**	**% no**	***n***	
If not a fire door, does the interior door of the medical office require less than 5 lbs pressure to open?	75.9	24.1	3558	
Is there a clear space in the waiting area that is not in the path of travel for a wheelchair or scooter user to park and wait?	89.6	10.4	3991	
Is there a clear path through the medical office free of objects that a blind person with a cane may not detect?	91.1	8.9	3989	
Is there a lower counter or an alternative method for people to sign in or register? (36.1% lower counter; 62.9% alternative method)	99.0	1.0	3989	
Does the examination room door opening meet width and clear opening standards?	91.2	8.8	3991	
Does the examination room have a 60" radius or T-shaped space for wheelchair or scooter user to enter and turn?	84.1	15.8	3990	

**Toilet room elements**			
Overall rating of toilet room compliance with 10 access elements				
**No. of elements**	**%**			
0–3	2.8			
4–6	20.4			
7–9	53.3			
10	23.6			

If not a fire door, does the interior door to the restroom require less than 5 lbs pressure to open?		88.1	11.9	3744
Are there grab bars at the accessible toilet located to the wall behind and the side?		85.6	14.3	3991
Is the toilet paper dispenser with the accessible toilet mounted in a position to meet access standards?		63.7	36.3	3991

**Equipment presence or characteristic**					
Is there a height-adjustable examination table that lowers to 17–19 inches?			19.1	80.9	3991
	**% yes**	**% no**			
With the height adjustable table, is there space for a wheelchair or scooter user to park and transfer or be assisted? (*n*=726)	96.3	3.7			
With the height adjustable table, are there rails or other elements to assist transfer and support a person? (*n*=682)	33.1	66.9			
Is a lift available to assist staff with transfers?			5.9	94.1	3989
Is there a weight scale within the office to accommodate a wheelchair or scooter user?			10.9	89.1	3989

A minority of offices have accessible examination equipment. We find 19.1% have a height adjustable examination table, 10.9% have an accessible weight scale, and 5.9% a lift that can assist staff and patient with transfer to an examination table. Where there is an accessible examination table, examination rooms have sufficient space for a wheelchair or scooter user to park next to the table and transfer; only one-third of the tables have elements such as rails and supports that increase safety and ease of use. Some MMC plans have purchased accessible equipment for offices; our data do not indicate purchaser, but one plan's providers had a noticeably higher presence of accessible equipment.

Bivariate correlations (Table 2) show the largest correlation coefficients between the equipment elements. The presence of an accessible scale is positively correlated with a height adjustable examination table (*r*=0.386, *p*<0.01) and a lift (*r*=0.321, *p*<0.01). The architectural elements such as door weights, waiting area space, and the bathroom elements are generally uncorrelated, except for bathroom grab bars and the examination room door opening (*r*=0.276, *p*<0.01). A lowered reception counter shows modest correlation with correctly positioned restroom grab bars (*r*=0.219, *p*<0.01) and the presence of an adjustable examination table (*r*=0.267, *p*<0.01) and accessible scale (*r*=0.209, *p*<0.01).

**Table 2.** Pearson Correlations for Accessible Examination Rooms and Equipment

	**Interior office door <5 lbs**	**Waiting area space for wheelchair and scooter**	**Path of travel clear**	**Low sign in counter**	**Interior restroom door <5 lbs**	**Toilet grab bars wall and side**	**Toilet paper mounting meets standard**	**Examination room door opening meets standard**	**Turning radius examination room**	**Height adjustable examination table**	**Lift available to assist transfer**	**Accessible scale available in office**
Interior office door <5 lbs	1.00											
Waiting area space for wheelchair and scooter	0.069^*^	1.00										
	3558											
Path of travel clear	0.018	0.101^**^	1.00									
	3556	3989										
Low sign in counter	0.106^**^	0.150^**^	0.007	1.00								
	3557	3989	3988									
Interior restroom door <5 lbs	0.275^**^	0.111^**^	0.046^**^	0.036^*^	1.00							
	3414	3744	3742	3742								
Toilet grab bars wall and side	0.032	0.039^*^	0.025	0.219^**^	0.035^*^	1.00						
	3558	3991	3989	3989	3744							
Toilet paper mounting meets standard	0.128^**^	0.007	0.041^**^	0.165^**^	0.084^**^	0.218^**^	1.00					
	3558	3991	3989	3989	3744	3991						
Examination room door opening meets standard	0.044^**^	0.066^**^	0.058^**^	0.159^**^	0.045^**^	0.276^**^	0.147^**^	1.00				
	3558	3991	3989	3989	3744	3991	3991					
Turning radius examination room	0.043^*^	0.145^**^	0.115^**^	0.125^**^	0.038^*^	0.058^**^	0.075^**^	0.106^**^	1.00			
	3557	3990	3988	3988	3743	3990	3990	3990				
Height adjustable examination table	0.058^**^	0.055^**^	0.022	0.267^**^	−0.008	0.122^**^	0.106^**^	0.108^**^	0.079^**^	1.00		
	3558	3991	3989	3989	3744	3991	3991	3991	3990			
Lift available to assist transfer	0.067^**^	0.061^**^	−0.009	0.173^**^	0.052^**^	0.014	0.020	0.036^*^	0.070^**^	0.196^**^	1.00	
	3557	3989	3987	3987	3742	3989	3989	3989	3988	3989		
Accessible scale available in office	0.031	0.063^**^	−0.021	0.209^**^	0.001	0.062^**^	0.064^**^	0.051^**^	0.076^**^	0.386^**^	0.321^**^	1.00
	3558	3989	3987	3987	3744	3989	3989	3989	3988	3989	3987	

^**^ Two-tailed significant <0.01.

^*^ Two-tailed significant <0.05.

Coded 1=Yes; 0=No.

## Discussion and Conclusions

The findings suggest that within a medical practice's interior space, building elements such as door widths, door swing, reception space, and examination room size are likely to comply with the ADAAG, with problems mostly in bathrooms and door weights. Elements not basic to construction, such as a lowered reception counter, grab bars, and toilet paper dispenser placement meet the ADAAG less often. Accessible examination equipment is still infrequently present. Compared to 2006–2009, a larger percentage of sites have a height-adjustable examination table in 2013–2016 (19.1% compared to 8.4%), but this is a small percentage overall. The presence of accessible weight scales (10.9% compared to 3.6%) is three times larger, but still too small to be functionally meaningful. The correlation findings suggest that medical practices with one piece of accessible equipment are somewhat more likely to have other pieces, and slightly more likely to have accessible reception counters and bathrooms. It may be that sensitivity in one aspect of accessibility generates sensitivity to other elements. Whether due to perceived patient need or compliance concerns, the MMC plans also may facilitate the presence of accessible equipment.

Most concerning is the continuing low percentage of doctor offices with accessible examination equipment. The ADA requires access, but not specific equipment, and enforcement works through complaints, not proactive auditing. For this reason, the MMC plans' audits have the potential for impact, especially as these organizations increasingly post accessibility ratings online for patients. California has now mandated these audits.^17^ Other states could emulate this, expanding potential impact and data. Beyond the tie to Medicaid, there is a need to develop, test, and measure what combination of incentives and penalties can increase medical office disability accessibility.

## Abbreviations Used

ADAAG: ADA Accessibility Guidelines
MMC: Medicaid Managed Care

## References

[1] Lagu TI, Iezonni LI, Lindenauer PK The axes of access—improving care for patients with disabilities. N Engl J Med. 2014;370:1847–1851 24806165 10.1056/NEJMsb1315940

[2] Story MF, Schwier E, Kailes JI Perspectives of patients with disabilities on the accessibility of medical equipment: examination tables, imaging equipment, medical chairs, and weight scales. Disabil Health J. 2009;2:169–179 21122757 10.1016/j.dhjo.2009.05.003

[3] Drum C, McClain MR, Horner-Johnson W, et al. *Health Disparities Chartbook on Disability and Racial and Ethnic Status in the United Status. Institute on Disability*. Durham, NH: University of New Hampshire, 2011. Available at www.iod.unh.edu Accessed 725, 2018

[4] Mahmoudi E, Meade MA Disparities in access to health care among adults with physical disabilities: analysis of a representative national sample for a ten-year period. Disabil Health J. 2015;8:182–190 25263459 10.1016/j.dhjo.2014.08.007

[5] Krahn GW, Walker DK, Correa-De-Araujo R Persons with disabilities as an unrecognized health disparity population. Am J Public Health. 2015;105(Supplement 2):S198–S206 25689212 10.2105/AJPH.2014.302182 PMC4355692

[6] Kroll T, Jones GC, Kehn M, et al. Barriers and strategies affecting the utilisation of primary preventive services for people with physical disabilities: a qualitative inquiry. Health Soc Care Community. 2006;14:284–293 16787479 10.1111/j.1365-2524.2006.00613.x

[7] Bauer SE, Schumacher JR, Hall A, et al. Disability and physical and communication-related barriers to health care related services among Florida residents: a brief report. Disabil Health J. 2016;9:552–556 27101882 10.1016/j.dhjo.2016.03.001

[8] Morris MA, Maragh-Bass AC, Griffin JM, et al. Use of accessible examination tables in the primary care setting: a survey of physical evaluations and patient attitudes. J Gen Intern Med. 2017;32:1342–1348 28924919 10.1007/s11606-017-4155-2 PMC5698222

[9] Singer R, Dickman I, Rosenfeld A Increasing the Physical Accessibility of Health Care Facilities. CMS OMH Issue Brief. Washington, DC: CMS Office of Minority Health, 2017

[10] Breslin ML *Promoting Physical and Programmatic Accessibility in Managed Long-Term Services and Supports Programs. Community Living Policy Center*. San Francisco, CA: University of California San Francisco, 2017. Available at https://clpc.ucsf.edu Accessed 924, 2018

[11] Sanchez JB, Byfield G, Brown TT, et al. Perceived accessibility versus actual physical accessibility of healthcare facilities. Rehabil Nurs. 2000;25:6–9 10754921 10.1002/j.2048-7940.2000.tb01849.x

[12] Mudrick NR, Breslin ML, Liang M, et al. Physical accessibility in primary health care settings: results from California on-site reviews. Disabil Health J. 2012;5:159–167 22726856 10.1016/j.dhjo.2012.02.002

[13] Story MF, Kailes JI, Mac Donald C The ADA in action at health care facilities. Disabil Health J. 2010;3:245–252 21122793 10.1016/j.dhjo.2010.07.005

[14] Graham CL, Mann JR Accessibility of primary care physician practice sites in South Carolina for people with disabilities. Disabil Health J. 2008;1:209–214 21122731 10.1016/j.dhjo.2008.06.001

[15] Lagu T, Hannon NS, Rothberg MB, et al. Access to subspecialty care for patients with mobility impairment. Ann Intern Med. 2013;158:441–446 23552258 10.7326/0003-4819-158-6-201303190-00003

[16] Pharr J Accessible medical equipment for patients with disabilities in primary care clinics: why is it lacking? Disabil Health J. 2013;6:124–132 23507163 10.1016/j.dhjo.2012.11.002

[17] State of California Health and Human Services Agency. Policy Letter 12-006 Revised Facility Site Review Tool. Department of Health Care Services Medi-Cal Managed Care Division. Sacramento, CA, 2012 Available at www.dhcs.ca.gov/formsandpubs/Documents/MMCDAPLsandPolicyLetters/PL2012/PL12-006.pdf Accessed 314, 2019

